# Supplementary material for: Patterns of Intron Gain and Loss in Fungi
Source: PLoS Biol. 2004 Nov 30;2(12):e422. doi: 10.1371/journal.pbio.0020422 (PMC532390; doi:10.1371/journal.pbio.0020422)
Supplement: Table S1 — Also available at http://genes.mit.edu/NielsenEtAl/. (4.3 MB ZIP). [file pbio.0020422.st001.zip › NielsenEtAl/html/184.html]

AN0305.1.NCU03051.1.MG04746.1.FG07055.1


```
 CLUSTAL W (1.82) Multiple Sequence Alignments - Introns Inserted


Sequence 1: NCU03051.1	964 aa
Sequence 2: FG07055.1	960 aa
Sequence 3: MG04746.1	968 aa
Sequence 4: AN0305.1	963 aa
Alignment Length: 979 aa
Number Identitical Residues: 490 aa
Alignment Score (without introns) 25476


MG04746.1 	MVKSYL2KYEHSKSFGLVSSSTSNIVWVG-NESKG----TGAGRVVTAANEEVLCWDVKK
NCU03051.1	MVKSYL2KFEPSKSFGVVATSSSNLVWTSSKDKSG----TGAGQAIVAANNEVLTWDIKK
FG07055.1 	MVKSYL2KYEHGKSFGVVASPTSNLVWSS-KDRTG----TSAGQAIVAANEEVICWDIKK
AN0305.1  	MVKSYL2KFEHSKTFGVITSASSNAIWVRDDAIAGISRQTGAGRSVVGAGEEVLCWDIKK
          	****** *:* .*:**::::.:** :*  ..   * : .*.**: :..*.:**: **:**

MG04746.1 	GELLSRWQDEKSKAQVTAIAQSKTDRDVFAVG~YEDGSIRLWDSKIATIIVSFNGHRTAI
NCU03051.1	GELLNRWKDENCKAQVTAIAQSKTDPDVFAVG~YEDGSIRLWDSKISTTIVSFNGHKSAI
FG07055.1 	GELISRWRDERCTVPVTAIAQSKADKDVFAVG~YEDGSIRLWDSKISSVILNFNGHKSAI
AN0305.1  	GELLGRWKDSACRAQVTVITQSKTDEDIFAVG2YEDGSIRLWDSRTETVMISFNGHKSAI
          	***:.**:*. . . **.*:***:* *:**** ***********:  : ::.****::**

MG04746.1 	THLAFDKSGTRLASGSKDTDVIVWDLVAEVGQYKLRGHKDQITGLCFIEPEPQTAEQEDA
NCU03051.1	TILAFDKSGVRLASGAKDTDVIVWDLVAEVGQYKLRGHKDQITGLRFIEPEPQIPEG-GD
FG07055.1 	TKLVFDKSGVRLASGSRDTDIIMWDLVAEVGQFKLRGHKDQITGLQFVEPEAQVQD---E
AN0305.1  	TQLAFDNAGVRLASGSRDTNIILWDLISEVGLFKLRGHTDQITSLHFLVPTLELLN----
          	* *.**::*.*****::**::*:***::*** :*****.****.* *: *  :  :    

MG04746.1 	EEAQAMVVDNEGS-EGFLLTTAKDSLIKLWDLSSRFCIETHVAQTNGECWALGVNPDFSC
NCU03051.1	DEEQAMVLDNEAT-EGFLLTTGKDALIKLWDLSSRHCIETHVAQNSGECWALGVSPDLSG
FG07055.1 	DGSRAMVIDGEGTHEGFLLTTGKDSLIKLWDLSSRHCIETHISQTNGECWALGLSPDYSG
AN0305.1  	-------EAGLSEHAGFLITTGKDALIKVWDLASQHCIETHIAQSNGECWSLGLAPDQSG
          	         . .   ***:**.**:***:***:*:.*****::*..****:**: ** * 

MG04746.1 	CVTAGNDGELKVWFIDLVALLSSTREVEVSHMGKILRDRGTLHRQSKDRAVEVRFHPRRD
NCU03051.1	CVTAGNDGEMRVWALDVAGLASSARKVDLSQAINFLHDRGTLHRSSKERAVEVIFHPRRD
FG07055.1 	CVTAGNDGEIKVWSLDADGLALSTRQVDAPAAAQYLQDKGTLHRQSKDRATEVVFHPKKD
AN0305.1  	CITGGNDGELKAWSIDEGAMIEISKEKTGSENRRILADRGSFYRNGKDRTTGISFHPRAD
          	*:*.*****::.* :*  .:   :::   .   . * *:*:::*..*:*:. : ***: *

MG04746.1 	YFAVHGIEKSVEIWRIRSGAEVKKALARKRKRRREKQGAKGDAEKDVKE--DADEAADDI
NCU03051.1	YFAVHGVEKSVEIWRIRNEAEIKKSLARKRRRRRELAAKKGKKEADAEE--EDDKA-DDI
FG07055.1 	YFAVHGSEKAVDVWRLRSEAEIKKALARKRRRRRE-KAKDGK-AAEEEA--NGEEP-EDV
AN0305.1  	YVAFHGSEKAVEIWRIRSETEVQKSLARKRKRRKEKEAQRAAETGETAEKTENGKAEDDV
          	*.*.** **:*::**:*. :*::*:*****:**:*  .  .    :   .::  :. :*:

MG04746.1 	SKADVSDIFVLLVIVRTTGKTRSVDWALTHGTKDLQLVVGTTNNQIELWHVTTTKDKEKK
NCU03051.1	SKADISDVFVQHVIVRTTGKVRSVAWALSQGTKDLQLLVGGTNNLLETYTIVG-KDK--L
FG07055.1 	SKADISDVFVQHVIVRTGGKVRSVDWAVPSGQKDLQLLVGTTNNLLEYYSVSR-KDKSDK
AN0305.1  	STAPVTEVFVSHTIVRTGGKVRSFDWIRTKSSGKIQLLAATTNNQLEAYSVTTLNKK---
          	*.* ::::**  .**** **.**. *  . .  .:**:.. *** :* : :   :.*   

MG04746.1 	SKKENAEDYTRALSIELPGHRADIRAIALSSDDRMLATAANGSLKIWNVRTQACIRTFEC
NCU03051.1	KSKGDAPDYNKALAVELPGHRTDIRALSISSDDKMLASAANGSLKIWNIKTNTCIRTFEC
FG07055.1 	SKKEDAPEYTRALSVELPGHRTDIRALSLSSDDKMLASASNGNLKIWNVKTTACIRTFEC
AN0305.1  	SKSEGDVEYNRTLAIDIPGHRTDIRSVALSSDDRMLASASNGSLKVWNVRTQNCLRTLEC
          	... .  :*.::*::::****:***::::****:***:*:**.**:**::*  *:**:**

MG04746.1 	GYALCCAFLPGDKVVVVGTKTGELELFDVASTALLDSVTAHD-GAIWSLHVHPDGRSVVT
NCU03051.1	GYALCCSFLPGDKVVVVGTKEGELQLYDVASASLLESVNAHEGHAIWSLQVHPDGKSVVS
FG07055.1 	GYALCCAFLPGDKVVVVGTKSGELQLFDVASAALLDSVDAHE-KAIWSLSVHPNGQSVVS
AN0305.1  	GYALCSAFLPGDKIVVVGNKDGELEIFDIASSTLLDTIKAHD-GPVWSLQVHPDGKSLVS
          	*****.:******:****.* ***:::*:**::**::: **:  .:*** ***:*:*:*:

MG04746.1 	GSADKSAKFWDFRIVQEPVLGTTRTTPKLKLVQTRVLKVSDDILSLKFSPDAKMLAVALL
NCU03051.1	GGADKTAKFWDFKIVQEQVLGTSRTTPKLKLVQSKILKVSDDILSLRFSPDAKLLAVALL
FG07055.1 	GSADQTAKFWDFKIVQEEVLGTRRTTPKLKLVQSRTLKVSHDILSLKFSPDAKLLAVALF
AN0305.1  	GSADKSAKFWNFQVVQEEIPGTKRTTPRLRLVHTRTLKVSDDILSVRFSPDARLLAVALL
          	*.**::****:*::*** : ** ****:*:**::: ****.****::*****::*****:

MG04746.1 	DSTVKVFFADSLKLYLNLYGHKLPVLSMDISYDSKLIITSSADKNIKIWGLDFGDCHKSL
NCU03051.1	DSTVKVFFVDSLKLYLNLYGHKLPVLSMDISYDNKLIVTSSADKNIRIWGLDFGDCHKAL
FG07055.1 	DSTVKVFFVDSLKLYLNLYGHKLPVLSMDISYDSKMIVTSSADKNIRIWGLDFGDCHKAL
AN0305.1  	DNTVKVFFNDSLKLFLNLYGHKLPVLNMDISWDSKLIVTCSADKTVRLWGLDFGDCHKSF
          	*.****** *****:***********.****:*.*:*:*.****.:::**********::

MG04746.1 	FGHQDSVLQVAFIPHNAEGNGHHFFSTSKDRVIKYWDGDKFEQIQRLHGHHGEVWALAVS
NCU03051.1	FGHQDSILQVAFVPHNSDGNGHHFFSASKDRTIKYWDADKFEQIQRIDGHHGEIWAIAVA
FG07055.1 	YGHKDSILQVAFVPHNNDGNGHHFFSSSKDRTIRYWDGDKFEQIQRMDGHHGEVWAIAVS
AN0305.1  	LAHEDSIMAVAFVPSNNEGNGHNFFSVSKDRVIKYWDGDKFEHIQKLSGHHGEIWAMAIS
          	 .*:**:: ***:* * :****:*** ****.*:***.****:**:: *****:**:*::

MG04746.1 	HSGNFIVSASHDKSIRVWEETDEQIFLEEEKEREIEELYEKTLTTSLERDEDAENEGEGD
NCU03051.1	HSGQFLISASHDKSIRVWEETDEQIFLEEEREKEIEELYESTLTTSLEQDPDAEDEN--R
FG07055.1 	HSGNLLVSAGHDKSIRIWDETDEQIFLEEEREKEMEELYESTLTTSLERDADAQDEN--N
AN0305.1  	HTGEFIVTASHDKSIRTWEQTDEQLFLEEEREKELEEIYDNNLAASLEEEEGGADGEK-A
          	*:*:::::*.****** *::****:*****:*:*:**:*:..*::***.: .. :  .  

MG04746.1 	EVGAASKQTVETLMAGERIQEALELGLEDLNTVREWQQARKTTPNLAPPQRSPVFTALGD
NCU03051.1	EVGAASKQTVETLMAGERIAEALELGMADLNAIKEWEEAKQQNPNVAPPQRNVLFMALGN
FG07055.1 	EVGAASKQTSETLMAGERIAEALEMGMADLNLLKEYEEAKLTNPHAQPPQRNMVFLALGD
AN0305.1  	EAVDAGKQTTDTLMAGEKIMEALDLGMEDLEVMREWRVIKAKNPNAAPPARNPLYMALNN
          	*.  *.*** :******:* ***::*: **: ::*:.  :  .*:  ** *. :: **.:

MG04746.1 	ITAEAYVMSVLQKIKAAALHDALLVLPFAAVPALFAFVDIFARREMNTPLTCRILFFMLK
NCU03051.1	ISAEQHVMNVLQKIKASALHDALLVLPFATVPMLFTFLNIFAMRSMNIPLTCRILFFMLK
FG07055.1 	ITAEAYVLSVLQKIKASALHDALLVLPFSSIPILFTFLNIFAMRSMNMPLTCRILFFMLK
AN0305.1  	ISAEQHLLNVVQKIPPAALQDALLVLPFSKIPALFTFLNIWASREWNVPLTCRVLFFMLK
          	*:** :::.*:*** .:**:********: :* **:*::*:* *. * *****:******

MG04746.1 	AHHKQIVASRTMRAALDDIRESLRRSLKRQKDEMGYNIAALKVVGMQVRERSVKDYVDEN
NCU03051.1	THHKQIVASKTMRAMLDGIRTNLRGALKRQKDEMGYNVAALKVVGMQLQHKSVKDYVDEK
FG07055.1 	THHKQIVASRSMKVMLDGIRINLRASLKRQKDEMGVNIAALKVVGMQIRDKSIKDYVDEN
AN0305.1  	THHRQIVASKLMRPMLDSIRSSLRRVLTRQKDEMGFNLSALQFVGEQVKAHGTKDYIDEQ
          	:**:*****: *:  **.** .**  *.******* *::**:.** *:: :. ***:**:

MG04746.1 	-WDKGND-VQAARKRTFVDVS
NCU03051.1	-WEEENEENKAVKKRAFVQVS
FG07055.1 	-WEEEND-ARSAKKRTFVDVA
AN0305.1  	AWEEEQRTTGTGKKRVFSSIA
          	:*:: :    : :**.* .::
```
